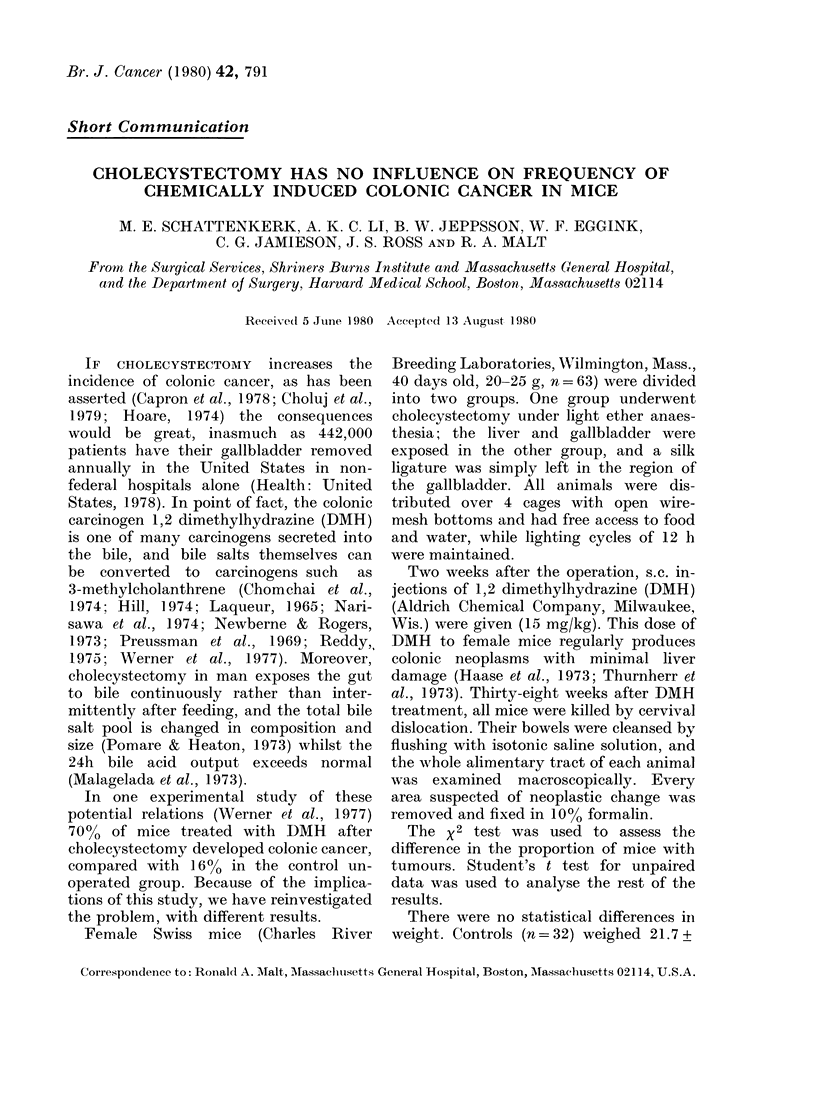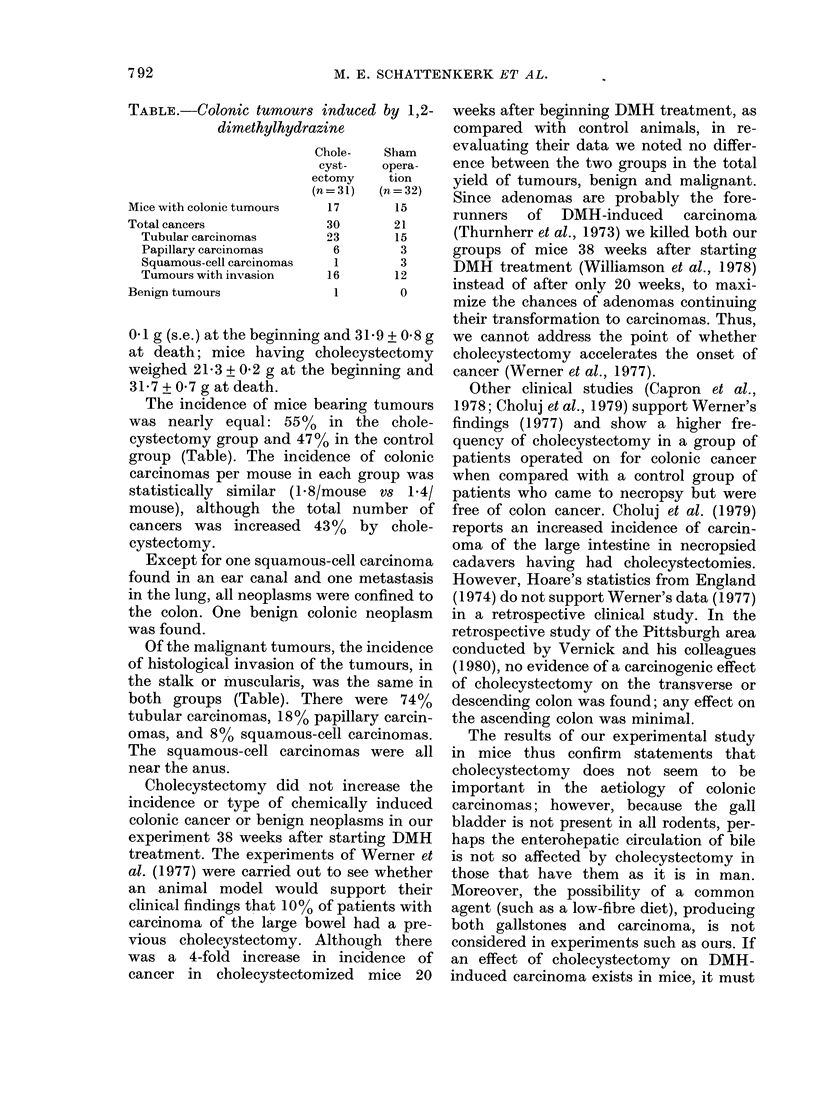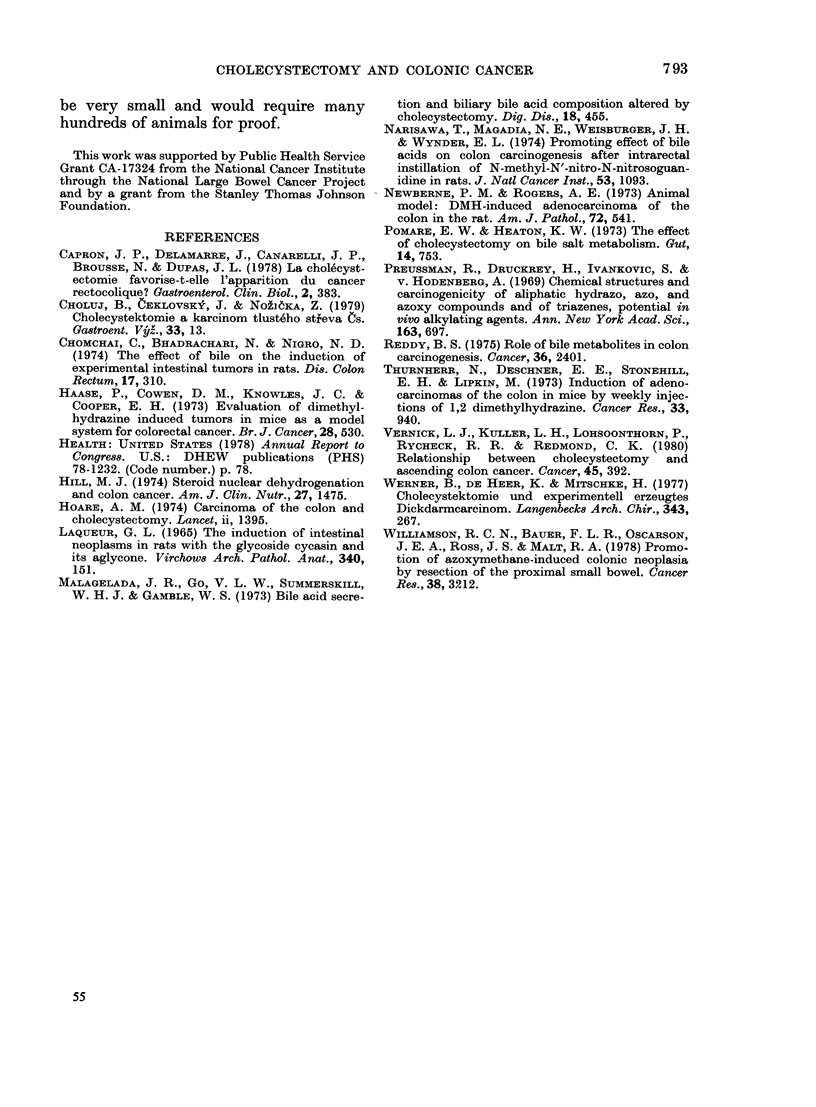# Cholecystectomy has no influence on frequency of chemically induced colonic cancer in mice.

**DOI:** 10.1038/bjc.1980.314

**Published:** 1980-11

**Authors:** M. E. Schattenkerk, A. K. Li, B. W. Jeppsson, W. F. Eggink, C. G. Jamieson, J. S. Ross, R. A. Malt


Br. J. Cancer (1980) **42**, 791

***Short Communication***

### CHOLECYSTECTOMY HAS NO INFLUENCE ON FREQUENCY OF CHEMICALLY INDUCED COLONIC CANCER IN MICE

M. E. SCHATTENKERK, A. K. C. LI, B. W. JEPPSSON, W. F. EGGINK, C. G. JAMIESON, J. S. ROSS AND R. A. MALT

*From the Surgical Services, Shriners Burns Institute and Massachusetts General Hospital, and the Department of Surgery, Harvard Medical School, Boston, Massachusetts* 02114

Received 5 June 1980 Accepted 13 August 1980

IF CHOLECYSTECTOMY increases the incidence of colonic cancer, as has been asserted (Capron *et al.*, 1978; Choluj *et al.*, 1979; Hoare, 1974) the consequences would be great, inasmuch as 442,000 patients have their gallbladder removed annually in the United States in non-federal hospitals alone (Health: United States, 1978). In point of fact, the colonic carcinogen 1,2 dimethylhydrazine (DMH) is one of many carcinogens secreted into the bile, and bile salts themselves can be converted to carcinogens such as 3-methylcholanthrene (Chomchai *et al.*, 1974; Hill, 1974; Laqueur, 1965; Narisawa *et al.*, 1974; Newberne & Rogers, 1973; Preussman *et al.*, 1969; Reddy, 1975; Werner *et al.*, 1977). Moreover, cholecystectomy in man exposes the gut to bile continuously rather than intermittently after feeding, and the total bile salt pool is changed in composition and size (Pomare & Heaton, 1973) whilst the 24h bile acid output exceeds normal (Malagelada *et al.*, 1973).

In one experimental study of these potential relations (Werner *et al.*, 1977) 70% of mice treated with DMH after cholecystectomy developed colonic cancer, compared with 16% in the control unoperated group. Because of the implications of this study, we have reinvestigated the problem, with different results.

Female Swiss mice (Charles River Breeding Laboratories, Wilmington, Mass., 40 days old, 20–25 g, $n = 63$) were divided into two groups. One group underwent cholecystectomy under light ether anaesthesia; the liver and gallbladder were exposed in the other group, and a silk ligature was simply left in the region of the gallbladder. All animals were distributed over 4 cages with open wire-mesh bottoms and had free access to food and water, while lighting cycles of 12 h were maintained.

Two weeks after the operation, s.c. injections of 1,2 dimethylhydrazine (DMH) (Aldrich Chemical Company, Milwaukee, Wis.) were given (15 mg/kg). This dose of DMH to female mice regularly produces colonic neoplasms with minimal liver damage (Haase *et al.*, 1973; Thurnherr *et al.*, 1973). Thirty-eight weeks after DMH treatment, all mice were killed by cervival dislocation. Their bowels were cleansed by flushing with isotonic saline solution, and the whole alimentary tract of each animal was examined macroscopically. Every area suspected of neoplastic change was removed and fixed in 10% formalin.

The $\chi^2$ test was used to assess the difference in the proportion of mice with tumours. Student's $t$ test for unpaired data was used to analyse the rest of the results.

There were no statistical differences in weight. Controls ($n = 32$) weighed 21.7 ±

Correspondence to: Ronald A. Malt, Massachusetts General Hospital, Boston, Massachusetts 02114, U.S.A.

TABLE.—*Colonic tumours induced by* 1,2-*dimethylhydrazine*

| | Cholecystectomy ($n=31$) | Sham operation ($n=32$) |
|---|---|---|
| Mice with colonic tumours | 17 | 15 |
| Total cancers | 30 | 21 |
| Tubular carcinomas | 23 | 15 |
| Papillary carcinomas | 6 | 3 |
| Squamous-cell carcinomas | 1 | 3 |
| Tumours with invasion | 16 | 12 |
| Benign tumours | 1 | 0 |

0·1 g (s.e.) at the beginning and 31·9 ± 0·8 g at death; mice having cholecystectomy weighed 21·3 ± 0·2 g at the beginning and 31·7 ± 0·7 g at death.

The incidence of mice bearing tumours was nearly equal: 55% in the cholecystectomy group and 47% in the control group (Table). The incidence of colonic carcinomas per mouse in each group was statistically similar (1·8/mouse *vs* 1·4/mouse), although the total number of cancers was increased 43% by cholecystectomy.

Except for one squamous-cell carcinoma found in an ear canal and one metastasis in the lung, all neoplasms were confined to the colon. One benign colonic neoplasm was found.

Of the malignant tumours, the incidence of histological invasion of the tumours, in the stalk or muscularis, was the same in both groups (Table). There were 74% tubular carcinomas, 18% papillary carcinomas, and 8% squamous-cell carcinomas. The squamous-cell carcinomas were all near the anus.

Cholecystectomy did not increase the incidence or type of chemically induced colonic cancer or benign neoplasms in our experiment 38 weeks after starting DMH treatment. The experiments of Werner *et al.* (1977) were carried out to see whether an animal model would support their clinical findings that 10% of patients with carcinoma of the large bowel had a previous cholecystectomy. Although there was a 4-fold increase in incidence of cancer in cholecystectomized mice 20 weeks after beginning DMH treatment, as compared with control animals, in re-evaluating their data we noted no difference between the two groups in the total yield of tumours, benign and malignant. Since adenomas are probably the forerunners of DMH-induced carcinoma (Thurnherr *et al.*, 1973) we killed both our groups of mice 38 weeks after starting DMH treatment (Williamson *et al.*, 1978) instead of after only 20 weeks, to maximize the chances of adenomas continuing their transformation to carcinomas. Thus, we cannot address the point of whether cholecystectomy accelerates the onset of cancer (Werner *et al.*, 1977).

Other clinical studies (Capron *et al.*, 1978; Choluj *et al.*, 1979) support Werner's findings (1977) and show a higher frequency of cholecystectomy in a group of patients operated on for colonic cancer when compared with a control group of patients who came to necropsy but were free of colon cancer. Choluj *et al.* (1979) reports an increased incidence of carcinoma of the large intestine in necropsied cadavers having had cholecystectomies. However, Hoare's statistics from England (1974) do not support Werner's data (1977) in a retrospective clinical study. In the retrospective study of the Pittsburgh area conducted by Vernick and his colleagues (1980), no evidence of a carcinogenic effect of cholecystectomy on the transverse or descending colon was found; any effect on the ascending colon was minimal.

The results of our experimental study in mice thus confirm statements that cholecystectomy does not seem to be important in the aetiology of colonic carcinomas; however, because the gall bladder is not present in all rodents, perhaps the enterohepatic circulation of bile is not so affected by cholecystectomy in those that have them as it is in man. Moreover, the possibility of a common agent (such as a low-fibre diet), producing both gallstones and carcinoma, is not considered in experiments such as ours. If an effect of cholecystectomy on DMH-induced carcinoma exists in mice, it must be very small and would require many hundreds of animals for proof.

This work was supported by Public Health Service Grant CA-17324 from the National Cancer Institute through the National Large Bowel Cancer Project and by a grant from the Stanley Thomas Johnson Foundation.

### REFERENCES

CAPRON, J. P., DELAMARRE, J., CANARELLI, J. P., BROUSSE, N. & DUPAS, J. L. (1978) La cholécystectomie favorise-t-elle l'apparition du cancer rectocolique? *Gastroenterol. Clin. Biol.*, **2**, 383.

CHOLUJ, B., ČEKLOVSKÝ, J. & NOŽIČKA, Z. (1979) Cholecystektomie a karcinom tlustého střeva Čs. *Gastroent. Výž.*, **33**, 13.

CHOMCHAI, C., BHADRACHARI, N. & NIGRO, N. D. (1974) The effect of bile on the induction of experimental intestinal tumors in rats. *Dis. Colon Rectum*, **17**, 310.

HAASE, P., COWEN, D. M., KNOWLES, J. C. & COOPER, E. H. (1973) Evaluation of dimethylhydrazine induced tumors in mice as a model system for colorectal cancer. *Br. J. Cancer*, **28**, 530.

HEALTH: UNITED STATES (1978) *Annual Report to Congress*. U.S.: DHEW publications (PHS) 78-1232. (Code number.) p. 78.

HILL, M. J. (1974) Steroid nuclear dehydrogenation and colon cancer. *Am. J. Clin. Nutr.*, **27**, 1475.

HOARE, A. M. (1974) Carcinoma of the colon and cholecystectomy. *Lancet*, ii, 1395.

LAQUEUR, G. L. (1965) The induction of intestinal neoplasms in rats with the glycoside cycasin and its aglycone. *Virchows Arch. Pathol. Anat.*, **340**, 151.

MALAGELADA, J. R., GO, V. L. W., SUMMERSKILL, W. H. J. & GAMBLE, W. S. (1973) Bile acid secretion and biliary bile acid composition altered by cholecystectomy. *Dig. Dis.*, **18**, 455.

NARISAWA, T., MAGADIA, N. E., WEISBURGER, J. H. & WYNDER, E. L. (1974) Promoting effect of bile acids on colon carcinogenesis after intrarectal instillation of N-methyl-N′-nitro-N-nitrosoguanidine in rats. *J. Natl Cancer Inst.*, **53**, 1093.

NEWBERNE, P. M. & ROGERS, A. E. (1973) Animal model: DMH-induced adenocarcinoma of the colon in the rat. *Am. J. Pathol.*, **72**, 541.

POMARE, E. W. & HEATON, K. W. (1973) The effect of cholecystectomy on bile salt metabolism. *Gut*, **14**, 753.

PREUSSMAN, R., DRUCKREY, H., IVANKOVIC, S. & v. HODENBERG, A. (1969) Chemical structures and carcinogenicity of aliphatic hydrazo, azo, and azoxy compounds and of triazenes, potential *in vivo* alkylating agents. *Ann. New York Acad. Sci.*, **163**, 697.

REDDY, B. S. (1975) Role of bile metabolites in colon carcinogenesis. *Cancer*, **36**, 2401.

THURNHERR, N., DESCHNER, E. E., STONEHILL, E. H. & LIPKIN, M. (1973) Induction of adenocarcinomas of the colon in mice by weekly injections of 1,2 dimethylhydrazine. *Cancer Res.*, **33**, 940.

VERNICK, L. J., KULLER, L. H., LOHSOONTHORN, P., RYCHECK, R. R. & REDMOND, C. K. (1980) Relationship between cholecystectomy and ascending colon cancer. *Cancer*, **45**, 392.

WERNER, B., DE HEER, K. & MITSCHKE, H. (1977) Cholecystektomie und experimentell erzeugtes Dickdarmcarcinom. *Langenbecks Arch. Chir.*, **343**, 267.

WILLIAMSON, R. C. N., BAUER, F. L. R., OSCARSON, J. E. A., ROSS, J. S. & MALT, R. A. (1978) Promotion of azoxymethane-induced colonic neoplasia by resection of the proximal small bowel. *Cancer Res.*, **38**, 3212.